# Spatiotempo﻿ral evolution analysis of NO_2_ column density before and after COVID-19 pandemic in Henan province based on SI-APSTE model

**DOI:** 10.1038/s41598-021-97745-y

**Published:** 2021-09-20

**Authors:** Yang Liu, Jinhuan Zhao, Kunlin Song, Cheng Cheng, Shenshen Li, Kun Cai

**Affiliations:** 1 Henan Engineering Laboratory of Spatial Information Processing, Henan Key Laboratory of Big Data Analysis and Processing, School of Computer and Information Engineering, Henan University, Kaifeng, 475004 People’s Republic of China; 2 State Key Laboratory of Remote Sensing Science, Aerospace Information Research Institute, Chinese Academy of Sciences, Beijing, 100101 People’s Republic of China; 3 School of Information and Electronics, Beijing Institute of Technology, Beijing, 100081 People’s Republic of China

**Keywords:** Atmospheric science, Environmental sciences

## Abstract

Air pollution is the result of comprehensive evolution of a dynamic and complex system composed of emission sources, topography, meteorology and other environmental factors. The establishment of spatiotemporal evolution model is of great significance for the study of air pollution mechanism, trend prediction, identification of pollution sources and pollution control. In this paper, the air pollution system is described based on cellular automata and restricted agents, and a Swarm Intelligence based Air Pollution SpatioTemporal Evolution (SI-APSTE) model is constructed. Then the spatiotemporal evolution analysis method of air pollution is studied. Taking Henan Province before and after COVID-19 pandemic as an example, the NO_2_ products of TROPOMI and OMI were analysed based on SI-APSTE model. The tropospheric NO_2_ Vertical Column Densities (VCDs) distribution characteristics of spatiotemporal variation of Henan province before COVID-19 pandemic were studied. Then the tropospheric NO_2_ VCDs of TROPOMI was used to study the pandemic period, month-on-month and year-on-year in 18 urban areas of Henan Province. The results show that SI-APSTE model can effectively analyse the spatiotemporal evolution of air pollution by using environmental big data and swarm intelligence, and also can establish a theoretical basis for pollution source identification and trend prediction.

## Introduction

With the society development, people pay more and more attention to the content change of various pollutants in the atmosphere, which greatly affects the change of global climate and atmospheric environment^1^. Air pollution is the result of comprehensive evolution^2^ of a complex and dynamic system^3^ composed of emission sources, topography, meteorology and other environmental factors. It is of great significance to build a spatiotemporal evolution model of air pollution to study the mechanism, prediction, pollution sources identification and treatment of air pollution^4^.

According to medical research, nitrogen dioxide (NO_2_) can stimulate the lungs, cause structural changes in the lungs, and make people more difficult to resist respiratory diseases such as cold, flu, and pneumonia. In early 2020, the Coronavirus Disease 2019 (COVID-19) pandemic disaster broke out all over the world. Some studies have found that prolonged exposure to high concentrations air pollution in patients with COVID-19 may increase mortality and vulnerability^5^. However, the global scientific community is still debating whether air pollution is related role to the mortality rates of COVID-19 and spread of the SARS-CoV-2 virus^6^. Although the pandemic has seriously affected the normal operation of the economy and life orders, it also provides unprecedented opportunities for Earth observation and atmosphere research. The sources of nitrogen oxides (NO_x_ = NO + NO_2_) in the atmosphere mainly includes natural and anthropogenic sources, and anthropogenic sources are the main sources of NO_2_ in the atmosphere. The study on the tropospheric NO_2_ Vertical Column Densities (VCDs) in China and some areas before and after the pandemic is of great significance for finding the internal mechanism of air pollution, and also provides data support and theoretical basis for air pollution control and prevention measures.

At present, there are two mains monitoring methods of NO_2_ in the atmosphere: ground station measurement and satellite remote sensing observation. Among them, the concentrations data obtained from the ground station measurement have the characteristics of high accuracy and all-weather. However, China's ground measurement stations were established in 2013. This traditional measurement method can only be carried out in a limited number of ground stations, and the distribution of stations is sparse. Satellite remote sensing observation has the advantages of wide coverage, providing macro change information, reflecting the large-scale of pollutants, etc., which can make up for the lack of observation spatial distribution of ground stations^7^.

In recent years, some scholars have used satellite remote sensing tropospheric VCDs inversion products to study the spatiotemporal evolution law of NO_2_ in atmospheric. Document^8^ studied long-term trends in tropospheric NO_2_ VCDs percentiles over Northeast Asia retrieved from the OMI (Ozone Monitoring Instrument) from 2005 to 2018, and show a significant increase in the background concentrations of tropospheric NO_2_ due to intensive industrial activities in Northeast Asia. Document^9^ researched temporal and spatial characteristics of the tropospheric NO_2_ VCDs by inversion data of OMI satellite remote sensing in the Sichuan Basin from 2005 to 2016. It is found that inter annual variation and spatial distribution trend of troposphere NO_2_ VCDs and nitrogen emission intensities are consistent. The spatial distributions of the NO_2_ VCDs were also affected by terrain, meteorological factors and its lifetime^10,11^. Based on the combination of tropospheric VCDs of NO_2_ and SO_2_ derived from OMI satellite with the ground station observations, Document^12^ studied analysed the spatiotemporal distribution of NO_2_ and SO_2_ amount in Inner Mongolia urban agglomerations. It finds that the diurnal variation of NO_2_ and SO_2_ is highly related to the diurnal variation of both anthropogenic emission and boundary layer height. However, most NO_2_ VCDs of satellite data are inversion products based on Differential Optical Absorption Spectroscop (DOAS) algorithm. The NO_2_ concentration is affected by cloud, a-priori NO_2_ profiles, aerosol layer, and other uncertain factors. There are systematic errors between single satellite sensor data and ground-based measurements data^13^.

In this paper, air pollution is described systematically based on Cellular Automata (CA) and Restricted Agent (RA), and constructs a Swarm Intelligence based Air Pollution SpatioTemporal Evolution (SI-APSTE) model. The troposphere NO_2_ VCDs products of TROPOMI and OMI were analysed based on the SI-APSTE model, and the NO_2_ variations in COVID-19 pandemic stage in Henan province were studied by TROPOMI products.

## Materials and method

### Overview of the study area

**Figure 1:**
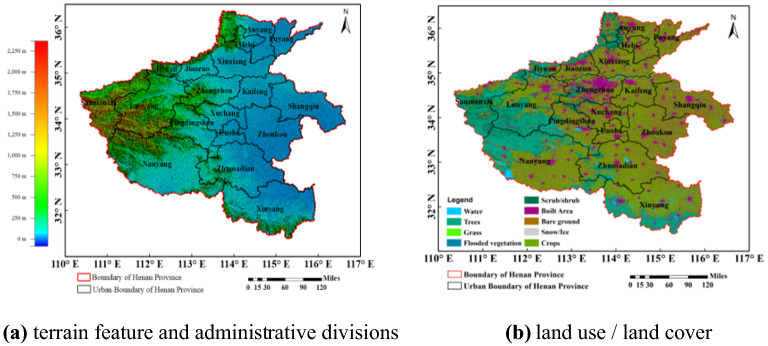
Terrain feature, administrative divisions, and land use / land cover of Henan Province.

Central Plains Economic Region (CPER) is located in the centre of China. The large-scale central plains urban is the most densely populated agglomerations in China at present. It is also the hub and core area to undertake industrial transfer and resource output, which has an important both strategically and economically. However, with the development of industrialization and urbanization, the air pollution of CPER becomes more and more serious. To study the spatiotemporal evolution of NO_2_ pollution in Henan Province can provide support of data and theoretical for pollution control and policy-making in the CPER. Henan Province is located at 31°23′ ~ 36°22′N, 110°21′ ~ 116°39′E, with a total area of 167,000 square kilometers. However, the population density is large, and the permanent population is about 94 million, accounting for 7.02% of the total permanent population of China. It belongs to subtropical and temperate monsoon climate with obvious seasonal changes. It is surrounded by mountains in the north, west and south, and plain in the central and eastern. The province consists of 17 prefecture level cities of Zhengzhou (ZZ), Kaifeng (KF), Luoyang (LY), Nanyang (NY), Luohe (LH), Xuchang (XC), Sanmenxia (SMX), Pingdingshan (PDS), Zhoukou (ZK), Zhumadian (ZMD), Xinxiang (XX), Hebi (HB), Jiaozuo (JZ), Puyang (PY), Anyang (AY), Shangqiu (SQ), Xinyang (XY) and Jiyuan (JY) and one county-level city directly under the provincial government. Figure 1 shows the terrain feature, administrative division, and land use/land cover of Henan Province.

### Data description

#### Satellite-based data collection and data pre-processing

Aura satellite, which was successfully launched on July 15, 2004, is a near polar satellite, which follows a sun-synchronous orbit with an equator crossing time near 13:45 local time. OMI measures the backscattered radiation from the sun using spectral bands ranging from the ultraviolet (UV) to infrared wavelengths. The OMI sensor on board mainly includes O_3_, NO_2_, SO_2_, HCHO and other gas products, among which the NO_2_ inversion uses DOAS algorithm^14^. The orbit scanning width of OMI sensor is 2600 km, and the spatial resolution of the sub-satellite point is 13 km × 24 km, which can cover the whole world once a day^15^. The comparison data used in this paper is the monthly mean density data of the tropospheric NO_2_ VCDs of OMI, which is from Tropospheric Emission Monitoring Internet Service (TEMIS) of the Royal Institute of Meteorology of the Netherlands (http://www.temis.nl/airpollution/no2col/no2regioomimonth_qa.php). The time span is from September 2018 to August 2019, a total of one-year data. The spatial resolution is 0.125° × 0.125°, and the unit is mol/cm^2^ (NO_2_ molecules per square centimeter). Then, the average VCDs were calculated on a quarterly and annual basis.

Sentinel-5p is a satellite dedicated to global air pollution monitoring launched by the European Space Agency (ESA) on October 13, 2017. Its TROPOspheric Monitoring Instrument (TROPOMI) imaging range is 2600 km, which can cover all parts of the world every day, and the imaging resolution is 7 km × 3.5 km. It can effectively monitor NO_2_, O_3_, SO_2_, CO and CH_4_ of global gases^16^. The observation equipment of Sentinel series satellite combines the advantages of other sensors and advanced technology, and has better observation performance. Compare with the Earth Observation System (EOS) of National Aeronautics and Space Administration (NASA) series satellite, its performance is greatly improved. Compared with other remote sensing instruments such as the Moderate Resolution Imaging Spectro-radiometer (MODIS), the Ozone Mapping and Profiler Suite (OMPS)^17^, the Ozone Monitoring Instrument (OMI) and Environmental Trace Gases Monitoring Instrument (EMI)^18^, TROPOMI can directly monitor the air pollution in small areas, and it can directly monitor the atmospheric pollution in small areas^16^. All TROPOMI data used in this paper are from the official NO_2_ data product of Royal Netherlands (http://www.temis.nl/airpollution/no2col/no2regio_tropomi.php).

#### In-situ data collection and data pre-processing

Research on OMI NO_2_ data has been relatively mature^19,20^, in order to further verify the universality of TROPOMI NO_2_ data, this paper uses a monthly ground-based measurements data from the national urban air quality real-time release platform of China Environmental Monitoring Centre (http://106.37.208.233:20035/) and tropospheric NO_2_ VCDs of TROPOMI in Henan Province were calculated. The date interval selected the data from the early stage of the pandemic from September 2018 to August 2019 and compared with the data of OMI sensor. Based on the comparison results and the advanced technology, the tropospheric NO_2_ VCDs in the previous month and the pandemic period (from December 21, 2019 to February 20, 2020) were selected to carry out the distribution of NO_2_ concentrations in Henan province for further research and analysis. Then, monthly, quarterly and annual average maps of NO_2_ VCDs are obtained by averaging.

### Air pollution system description based on cellular automata and restricted agent

The generation of air pollution events is a dynamic evolution product of complex system composed of emission sources, topography, meteorology and other environments. In this paper, cellular automata (CA) and restricted agent (RA) are used to describe the dynamic variation process of air pollutant concentrations in a specific spatiotemporal research area. CA is a kind of grid dynamic model with local spatial interaction and temporal causality in discrete spatiotemporal state, which has the ability to simulate the dynamic spatiotemporal evolution process of complex system. CA has unique advantages in modelling pollution system with hydrodynamic characteristics^21–25^. We consider that each pixel of the pollutant concentration map is composed of a CA. The actual concentration contribution of each pixel (described by CA) comes from the fixed source described by RA and the mobile source described by Mobile Agent (MA) respectively. For the concentration change of pollutant gase (such as NO_2_, SO_2_, O_3_, CO and CH_4_) in a specific space–time, the CA(*s, t*) system is described as:

1 $$\begin{array}{*{20}c} {{\text{CA(}}s,t{)} = \left( {L,d,D,N,{\text{F}} } \right),} & {s.t.} & {{\text{F}}:} \\ \end{array} D_{i}^{t + 1} = {\text{F}} \left( {D_{{\text{i}}}^{t} ,D_{N}^{t} } \right),$$

where *s* is the space of cellular CA, *t* is the time of cellular CA, L is the cell neighbourhood space, and L can adopt von Neuman type of 4-neighborhood^26^, Moore type of 8-neighborhood^27^ or Margolus neighbourhood^28^; d is the dimension of cellular CA; D is the finite and discrete set of pollution state of cellular, and the state of D is determined by the concentrations of pollutants; N refers to the set of all cellular in a neighbourhood; F refers to the local mapping or local rules, indicating the emission, migration, diffusion and degradation of pollutants in cellular CA.

In the field of distributed system of artificial intelligence, agent is an abstract entity which has independent thinking and can interact with environment independently^29^. The restricted agent RA (*s, t*) is used to describe the dynamic emission objects of the pollution source, and RA is described by triples:

2 $$\begin{array}{*{20}c} {{\text{RA(}}s,t{)} = \left( {\text{C,R,Z}} \right),} & {\begin{array}{*{20}c} {s.t.} & {{\text{Z}}:{\text{C}}(s,t) \to {\text{R}}} \\ \end{array} } \\ \end{array},$$

where *s* is the current location of the RA, *t* is the time of the RA, *C* is the current perceived environment, *R* is the emission of pollutants (such as NO_x_^30^, SO_2_, CO^31^, NH_3_, O_3_, VOC, etc.), and the mapping Z meets Z: *C* (*s, t*) → *R*. There are restrictions on RA spatiotemporal information and message delivery. There is no information transfer in RA. The emission pollutants of RA directly control the pollution state of cellular CA in the same location. For a fixed pollution source, the location *s* of the agent RA is fixed; for a mobile pollution source, the agent RA can be described by the MA, and the spatiotemporal location (*s, t*) of MA is simulated by one-dimensional cellular according to its moving path.

### Swarm intelligence based air pollution spatiotemporal evolution model

**Figure 2:**
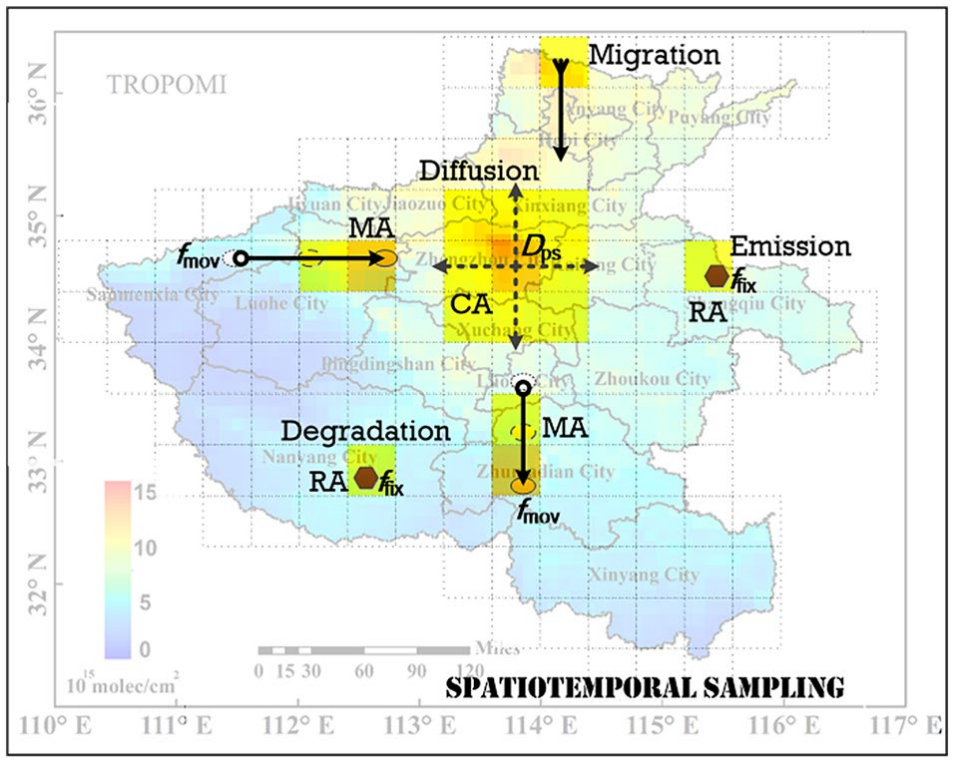
The pollutants emission, migration, diffusion and degradation between CA, MA and RA in SI-APSTE model.

The Air Pollution System (APS) described by CA and RA can be used to build a Swarm Intelligence based Air Pollution SpatioTemporal Evolution (SI-APSTE) model (Fig. 2). The CA model can be established by using historical environmental data, which can further realize the pollution inversion, prediction and simulation of the APS. Swarm intelligence (SI) is a bionic computing method in the field of artificial intelligence^32^. Through interaction and cooperation among micro low-level intelligence individuals, SI shows collective intelligence behaviour at the macro high-level. The spatiotemporal evolution of air pollution is similar to the generation, migration, aggregation and dissipation of particle swarm. Combining with the SI algorithm such as Ant Colony Optimization (ACO) and Particle Swarm Optimization (PSO)^33,34^, the RA objects that emission pollution can be reversely inferred according to the CA pollution state, so as to achieve the purpose of pollution sources identification. The pollutant concentrations D_PS_(*s, t*) of cellular CA(*s, t*) meet the following requirements:

3 $${\text{D}}_{{{\text{ps}}}} {(}s,t{\text{) = D}}_{{{\text{em}}}} {(}s,t{\text{) + D}}_{tr} {(}s,t{\text{) + D}}_{ac} {(}s,t{)},$$

Here, D_em_(*s, t*) is the emission pollutant concentration of the agent RA in the cellular CA(*s, t*), D_tr_(*s, t*) concentration is the pollutant migration of the cellular CA(*s, t*), and D_ac_(*s, t*) is the pollutant accumulated concentration of the cellular CA(*s, t*).

In essence, both satellite remote sensing and ground station measurement are a kind of spatiotemporal sampling of on-site pollution data. According to the principle of information theory, only when the measurement time interval and spatial resolution meet the sampling theorem can the real pollution situation be reflected. So we use Nyquist–Shannon sampling theorem^35,36^ to describe the authenticity of pollution information in SI-APSTE model. According to So SI-APSTE can be generated by using CA dynamic complex system and environmental historical data based on spatiotemporal sampling. Generally, the measured pollution data of the ground station belongs to oversampling in time, but due to the sparsity of its distribution, it is generally undersampling in space. On the contrary, for the satellite remote sensing inversion of pollutant data, due to the limitation of satellite transit revisit period, it is often undersampling in time, but generally has a relatively high resolution in space. By using the methods of information fusion, downsampling interpolation and subsampled, the multi-source data of multiple satellite observations^37^ and ground-based data can be fused to generate a high-precision and effective spatiotemporal evolution model of air pollution.

Generally, there are two sources of air pollutant emission concentration D_em_(*s, t*) of cellular CA(*s, t*): fixed emissions source RA pollutant *f*_fix_ (*s, t*) and mobile emissions source MA pollutant *f*_mov_ (*s, t*):

4 $${\text{D}}_{em} {(}s,t{) = }\begin{array}{*{20}c} {f_{{{\text{f}}ix}} {(}s,t{)} + f_{mov} {(}s,t{),}} & {\begin{array}{*{20}c} {s.t.} & {f_{{{\text{f}}ix}} {(}s,t{)} \propto N_{eco} {(}s,t{)},\;\;\;f_{mov} {(}s,t{)} \propto N_{{{\text{tfc}}}} {(}s,t{)}} \\ \end{array} } \\ \end{array},$$

Here, *f*_fix_ (*s, t*) is correlated with local economic environment *N*_eco_(*s, t*)^38^, *f*_mov_ (*s, t*) is correlated with local traffic environment *N*_*tfc*_(*s, t*)^39^. *D*_*tr*_(*s, t*) of air pollutants concentration migration and diffusion in cellular CA(*s, t*) meets the following requirements:

5 $${\text{D}}_{tr} {(}s,t{) = }\left( {f_{trI} {(}s,t{) + }f_{diI} {(}s,t{)}} \right){ - }\left( {f_{trO} {(}s,t{) + }f_{diO} {(}s,t{)}} \right),\;\;\;\;s.t.\;\;\;\;\;\left\{ \begin{gathered} f_{diO} {(}s,t{)} \in {\text{D}}_{{{\text{ps}}}} {(}s,t_{0} {)}Gauss{(}s,t{)} \hfill \\ f_{diI} {(}s,t{)} \in {\text{D}}_{{{\text{ps}}}} {(}s,t_{0} {)}\left( {{1 - }Gauss{(}s,t{)}} \right) \hfill \\ f_{trI} {(}s,t{)} \propto {\text{D}}_{{{\text{ps}}}} {(}s_{i} ,t_{j} {\text{)N}}\left( {W_{{{\text{mete}}}} {(}s,m{)},G_{{{\text{terr}}}} {(}s{)}} \right) \hfill \\ f_{trO} {(}s,t{)} \propto {\text{D}}_{{{\text{ps}}}} {(}s_{i} ,t_{j} {\text{)N}}\left( {W_{{{\text{mete}}}} {(}s,m{)},G_{{{\text{terr}}}} {(}s{)}} \right) \hfill \\ \end{gathered} \right.,$$

Among them, *f*_trI_(*s, t*) is the quantity of entering cellular pollutants by migration, *f*_trO_(*s, t*) is the quantity of exit cellular pollutants by migration, and the pollutants migration of CA(*s, t*) is related to the local meteorological *W*_mete_^40^ and terrain *G*_terr_^41^; *f*_diI_(*s, t*) is the amount of entering cellular CA(*s, t*) pollutant by diffusion, and *f*_diO_(*s, t*) is the amount of exit cellular CA(*s, t*) pollutant by diffusion, and the spatial–temporal diffusion of cellular CA(*s, t*) pollutant meets the Gaussian distribution characteristics^42^.

The accumulated amount of air pollutants in cellular CA(*s, t*), *D*_ac_(*s, t*), meets the following requirements:

6 $$\begin{gathered} {\text{D}}_{{{\text{ac}}}} {(}s,t{\text{) = D}}_{{{\text{ps}}}} {(}s,t_{0} {)} - f_{deg} {(}t{),}\;\;\;s.t.\;\;\;\;f_{deg} (t) \propto N\left( {W_{temp} (m),W_{humi} (m),W_{{{\text{v}}ege}} (m)} \right) \hfill \\ \hfill \\ \end{gathered} ,$$

Here, the amount of degradation *f*_deg_(*t*) is the amount of pollutant migration, and the amount of degradation *f*_deg_(*t*) is related to the local atmospheric humidity *W*_humi_^43^, temperature *W*_temp_, and vegetation *W*_vege_^44^. D_ps_(*s, t*_*0*_) is air pollutant concentration of cellular CA(*s, t*).

### Analysis of the air pollution spatiotemporal evolution

According to the SI-APSTE model, the air pollutants of the cellular CA(*s, t*) meet the following reduction:

7 $$\begin{aligned} {\text{D}}_{{{\text{ps}}}} {(}s,t{)} & {\text{ = D}}_{em} {(}s,t{\text{) + D}}_{tr} {(}s,t{\text{) + D}}_{ac} {(}s,t{)} \\ & {\text{ = D}}_{em} {(}s,t{) + }f_{trI} {(}s,t{)} - f_{trO} {(}s,t{\text{) + D}}_{{{\text{ps}}}} {(}s,t_{0} {)}f_{diI} {(}s,t{)} - {\text{D}}_{{{\text{ps}}}} {(}s,t_{0} {)}f_{diO} {(}s,t{)} - f_{deg} {(}t{)} \\ & {\text{ = D}}_{em} {(}s,t{) + }f_{trI} {(}s,t{\text{) + D}}_{{{\text{ps}}}} {(}s,t_{0} {)}f_{diI} {(}s,t{)} - f_{trO} {(}s,t{)} - {\text{D}}_{{{\text{ps}}}} {(}s,t_{0} {)}f_{diO} {(}s,t{)} - f_{deg} {(}t{)} \\ & { = }\left( {f_{fix} {(}s,t{)} + f_{mov} {(}s,t{)}} \right){ + }\left( {f_{trI} {(}s,t{)} - f_{trO} {(}s,t{)}} \right){\text{ + D}}_{{{\text{ps}}}} {(}s,t_{0} {)}\left( {f_{diI} {(}s,t{)} - f_{diO} {(}s,t{)}} \right) - f_{deg} {(}t{)} \\ \end{aligned}$$

#### Temporal and spatial variance analysis of cellular pollution

The air pollutant concentration D_ps_(*s, t*) of cellular CA(*s, t*) at different time meets the following reduction:

8 $$\frac{{\partial {\text{D}}_{{{\text{ps}}}} {(}s,t{)}}}{{\partial {\text{t}}}} \approx \frac{{\partial {\text{D}}_{em} {(}s,t{)}}}{{\partial {\text{t}}}}{ = }\frac{{\partial f_{fix} {(}s,t{)}}}{{\partial {\text{t}}}}{ + }\frac{{\partial f_{mov} {(}s,t{)}}}{{\partial {\text{t}}}}$$

Theoretically, the time interval and spatial distance of pollution data measurement should be as small as possible. However, for satellite remote sensing, there is a limit of revisit period, which cannot be infinitely small (for satellites, the revisit period is usually days, and for ground stations, the measurement interval is usually in hours); for ground measurement, there is also a limit of station construction, which cannot be built anywhere. If there is no special case, when the time interval is small, the emission of fixed emission sources generally changes little. It can be considered that the change of pollution emission of fixed emission sources tends to 0, that is:

9 $$\frac{{\partial {\text{D}}_{{{\text{ps}}}} {(}s,t{)}}}{{\partial {\text{t}}}} \approx \frac{{\partial f_{mov} {(}s,t{)}}}{{\partial {\text{t}}}},\;\;\;s.t.\;\;\;\frac{{\partial f_{{{\text{fix}}}} {(}s,t{)}}}{{\partial {\text{t}}}} \to {0}$$

#### Temporal and spatial accumulation analysis of cellular pollution

The air pollutant concentration *D*_*ps*_(*s, t*) accumulation of cellular CA(*s, t*) at different time meets the following formula:

10 $$\sum {{\text{D}}_{{{\text{ps}}}} {(}s,t{)}} { = }\sum {{\text{D}}_{em} {(}s,t{)}} { + }\sum {{\text{D}}_{tr} {(}s,t{)}} { + }\sum {{\text{D}}_{ac} {(}s,t{)}}$$

In the long run, for the time cellular CA(*s, t*) meet the conservation of mass, because the diffusion of pollutants does not affect the change of the total amount of pollutants, the pollutants of the migration enter and exit will tend to balance:

11 $$\sum {{\text{D}}_{{{\text{ps}}}} {(}s,t{)}} \approx \sum {{\text{D}}_{em} {(}s,t{)}} { + }\sum {{\text{D}}_{ac} {(}s,t{)}} = \sum {{\text{D}}_{em} {(}s,t{)}} - \sum {f_{deg} } ,\;\;\;s.t.\;\;\;\sum {{\text{D}}_{{{\text{tr}}}} {(}s,t{)}} \to {0}$$

#### Multivariate analysis and correlation analysis of air pollution components

If the spatial–temporal differences of cellular are ignored, air pollution indices such as PM_2.5_ and PM_10_ can be described by the multiple pollutants in the multiple cellular system^45^. If the preparing the data follows the normal distribution, multiple linear regression and Pearson correlation analysis are used; On the contrary, non-parametric regression (such as kernel regression, multivariate adaptive regression splines) and Sperman correlation analysis are used. Ideally, multiple linear regression analysis is carried out for the air pollution composite index $\overline{\overline{{{\text{D}}_{ps}^{pm} }}}$ and each pollution component:

12 $$\overline{\overline{{{\text{D}}_{ps}^{pm} }}} { = }\beta_{0} { + }\sum\limits_{k = 1}^{N} {\left( {\beta_{k} \times f({\text{D}}_{ps}^{k} )} \right)} ,\;\;\;\;\;s.t.\;\;\;\;k \in \left\{ {NO_{2} ,SO_{2} ,NH_{3} ,O_{3} ,VOC, \cdots } \right\},\;\;\;R = \sqrt {\frac{{\sum\limits_{{{\text{i}} = 1}}^{n} {\left( {\overline{\overline{{{\text{D}}_{ps}^{pm} }}} \left( i \right) - \overline{{{\text{D}}_{ps}^{pm} }} } \right)^{2} } }}{{\sum\limits_{{{\text{i}} = 1}}^{n} {\left( {{\text{D}}_{ps}^{pm} \left( i \right) - \overline{{{\text{D}}_{ps}^{pm} }} } \right)^{2} } }}} ,\overline{{{\text{D}}_{ps}^{pm} }} { = }\frac{1}{{\text{n}}}\sum\limits_{{{\text{i}} = 1}}^{n} {{\text{D}}_{ps}^{pm} \left( i \right)} ,R \in \left[ {0,1} \right]$$

Here, ${\text{D}}_{ps}^{k}$ is the concentration of the k-th air pollutant. $\beta_{k}$ is the regression coefficient. N is the number of type pollutant component. n is the monitoring sample number of pollutant component. *R* is the multiple correlation coefficients. *R* reflects the linear dependence measurement or complex nonlinear dependence measurement between the total pollution index and each pollution component. When only one pollutant is considered, that is. N = 1 and ${\text{D}}_{ps}^{k} = f({\text{D}}_{ps}^{k} )$, the above Formula (12) is becoming a linear regression equation of one variable:

13 $$\begin{aligned} {\text{D}}_{ps}^{pm} & { = }\beta { + }r \times \alpha \times {\text{D}}_{ps} ,\;\;\;\;s.t.\;\;\;\;\;r \in \left[ { - 1,1} \right],\;\;\;\overline{{{\text{D}}_{ps}^{pm} }} { = }\frac{1}{{\text{n}}}\sum\limits_{{{\text{i}} = 1}}^{n} {{\text{D}}_{ps}^{pm} \left( i \right)} ,\;\;\;\overline{{{\text{D}}_{ps}^{{}} }} { = }\frac{1}{{\text{n}}}\sum\limits_{{{\text{i}} = 1}}^{n} {{\text{D}}_{ps}^{{}} \left( i \right)} , \\ \alpha & { = }\sqrt {\frac{{\sum\limits_{{{\text{i}} = 1}}^{n} {\left( {{\text{D}}_{ps}^{pm} (i) - \overline{{{\text{D}}_{ps}^{pm} }} } \right)} }}{{\sum\limits_{{{\text{i}} = 1}}^{n} {\left( {{\text{D}}_{ps}^{{}} (i) - \overline{{{\text{D}}_{ps}^{{}} }} } \right)} }}} ,r = \frac{{\sum\limits_{{{\text{i}} = 1}}^{n} {\left( {{\text{D}}_{ps}^{{}} (i) - \overline{{{\text{D}}_{ps}^{{}} }} } \right)\left( {{\text{D}}_{ps}^{pm} (i) - \overline{{{\text{D}}_{ps}^{pm} }} } \right)} }}{{\sqrt {\sum\limits_{{{\text{i}} = 1}}^{n} {\left( {{\text{D}}_{ps}^{{}} (i) - \overline{{{\text{D}}_{ps}^{{}} }} } \right)}^{2} \sum\limits_{{{\text{i}} = 1}}^{n} {\left( {{\text{D}}_{ps}^{pm} (i) - \overline{{{\text{D}}_{ps}^{pm} }} } \right)^{2} } } }} \\ \end{aligned}$$

Here *r* is a simple correlation coefficient, which can be used to test the effectiveness of satellite remote sensing inversion data with ground-based measured data, as well as the inversion accuracy of different satellite remote sensing data, which is a commonly used analysis method of ideal state minimalism. In fact, due to the influence of atmospheric physics, atmospheric chemistry, ecological environment and other factors, the pollution components often depend on each other and influence each other, showing a nonlinear relationship. For example, there is a complex nonlinear relationship between the pollutant concentration of ground level based on the in-situ ground station and the vertical column density of pollutant based on satellite inversion.

#### MoM and YoY rate analysis of cellular pollution

Processing and analysis the pollution monitoring data in different time and space can effectively reveal the differences and contradictions, and mining the changing trend of environmental big data. The month-on-month (MoM) rates used to compare pollution for one month with those in previous months. The calculation method of the MoM rate *DR*_*MoM*_(*s, t*) of CA(*s, t*) pollution is as follows:

14 $$DR_{MoM} (s,t) = \frac{{D_{Cps} (s,t) - D_{Lps} (s,d)}}{{D_{Lps} (s,d)}} \times 100\% ,\;\;\;\;\;\;s.t.\;\;\;\;\;d = t - T,T \in \left\{ {day, \;week, \;month, \;quarter, \;year} \right\}$$

Here, *D*_Cps_(*s, t*) is the pollution concentration in the current period *t*(a certain statistical period), D_Lps_(*s, t*) is the pollution concentration in the previous period *d*(the previous statistical period), and statistical period *t* can be day, week, month, quarter or year. In order to eliminate the impact of seasonal changes, a year-on-year (YoY) calculation compares a pollution concentration statistic for one period to the same period the previous year. The YoY rate of cellular CA(*s, t*) pollution *DR*_*YoY*_(*s, t*) is calculated as follows:

15 $$DR_{YoY} (s,t) = \frac{{D_{Cps} (s,t) - D_{{Y{\text{ps}}}} (s,y)}}{{D_{{Y{\text{ps}}}} (s,y)}} \times 100\% ,\;\;\;\;s.t.\;\;\;y = t - year$$

Here, *D*_*Cps*_(*s, t*) is the pollution concentration in the current period *t*, *D*_*Yps*_(*s, t*) is the pollution concentration in the last year the same period *y*.

## Results and discussion

SI-APSTE model would analyse the dynamic variations of pollutants in different regions and the monitoring effect of different atmospheric inversion products. It can also realize the pollution source identification and further carry out the prediction of pollution trend research. Considering that NO_2_ emission is the main factor affecting VCDs, we ignore the seasonal, lifetime and the impact of meteorology factors of NO_2_. Taking the monitoring of NO_2_ before and after the COVID-19 pandemic in Henan Province as an example, this paper analysis the inversion products of TROPOMI and OMI NO_2_, and studies the temporal and spatial evolution of NO_2_ based on SI-APSTE model.

In order to compare the monitoring of NO_2_ between TROPOMI and OMI sensors, the spatiotemporal distribution and variation of tropospheric NO_2_ VCDs of 18 cities in Henan Province from September 2018 to August 2019 (one year), as well as the annual spatial distribution is analysed. According to the results of the study, TROPOMI products with more advanced performance and higher spatial resolution were used to analyse the variation of NO_2_ VCDs during the pandemic in Henan Province.

### The NO_2_ VCDs spatiotemporal evolution analysis of monthly variation in early stage of COVID-19 pandemic in Henan province

**Figure 3:**
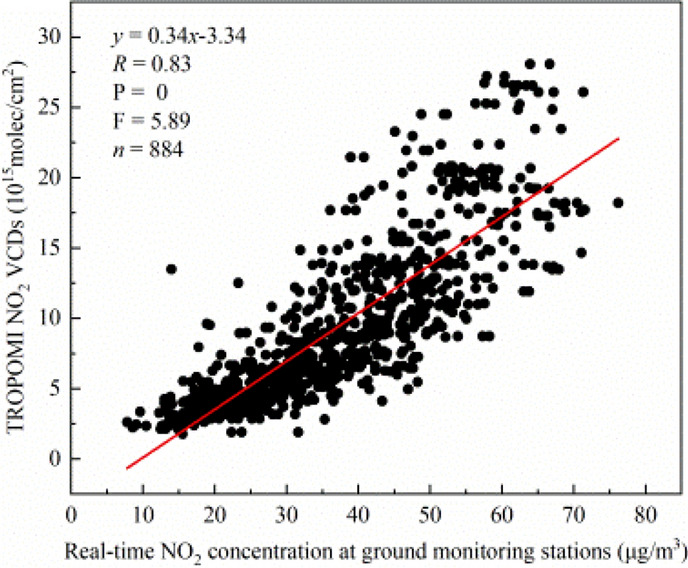
Correlation analysis of TROPOMI NO_2_ VCDs and real-time NO_2_ concentration at ground monitoring stations.

According to the Formula (13) of SI-APSTE model, the correlation of tropospheric NO_2_ VCDs between them is analysed. Among the 83 monitoring stations published on the platform from September 2018 to August 2019, 8 monitoring stations have no data, and the data of 3 monitoring stations are partially missing. Finally, 884 effective NO_2_ concentrations data and tropospheric NO_2_ VCDs of TROPOMI are selected for correlation analysis. As shown in Fig. 3, there is a significant positive correlation between the monthly average ground NO_2_ concentrations and the monthly average TROPOMI NO_2_ VCDs, with a correlation coefficient *R* of 0.83, T-test probability P-value = 0 and F-Test statistic F value = 5.89. It shows that the Sentinel-5P remote sensing satellite has a good monitoring ability for the NO_2_ emission on the ground in Henan Province. It can be very sensitive to monitor the emission level of industrial sources and urban traffic sources near the ground. It can provide strong support for the fine control of regional air pollution and the accurate early warning and prediction of air quality.

**Figure 4:**
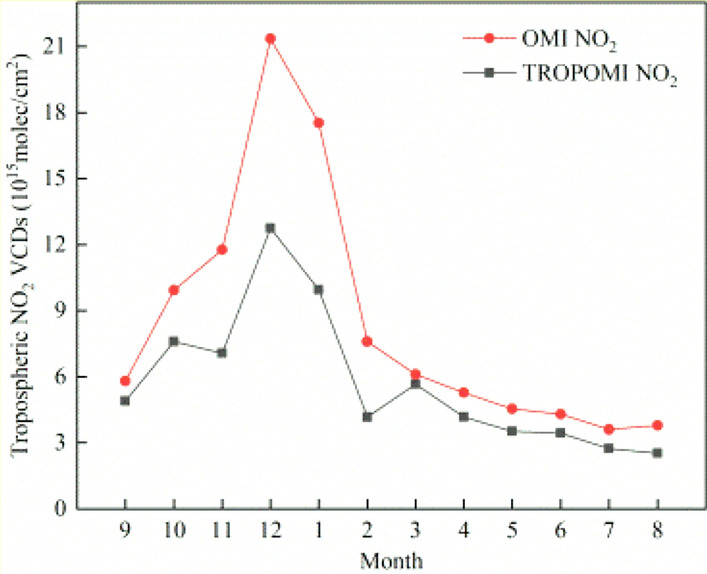
Monthly average trend of NO_2_ VCDs of the COVID-19 early period from September 2018 to August 2019 in Henan province.

In order to analyse the monthly spatial variation of NO_2_ VCDs in Henan Province, the monthly variation trend of NO_2_ concentration was studied in the early stage of the COVID-19 pandemic. The monthly variation NO_2_ concentration was obtained by the way of daily average accumulation (excluding 0 values) from September 2018 to August 2019 in Henan Province. As shown in Fig. 4, the NO_2_ VCDs trend of both TROPOMI and OMI is roughly the same, but the NO_2_ VCDs fluctuation of TROPOMI is more detailed than OMI. On the whole, the NO_2_ VCDs of OMI is higher than that of TROPOMI, especially in December; the difference reaches the maximum value of 8.62 × 10^15^ mol/cm^2^ in a year. The high values of both NO_2_ concentration occurred in December 2018, in which the maximum NO_2_ VCDs values of TROPOMI and OMI were 12.74 × 10^15^ mol/cm^2^ and 21.36 × 10^15^ mol/cm^2^ respectively. The low values occurred in July and August 2019, in which the minimum NO_2_ VCDs values of TROPOMI reached 2.52 × 10^15^ mol/cm^2^ in August, and the minimum NO_2_ VCDs values of OMI reached 3.61 × 10^15^ mol/cm^2^ in July. On the whole, the NO_2_ VCD retrieved from OMI is higher than TROPOMI. Explanations for this difference may result from the restrictions in the different inversion algorithms and satellite sensors (such as spatial resolution, spectral coverage, wavelength calibration et al.)^46,47^.

**Figure 5:**
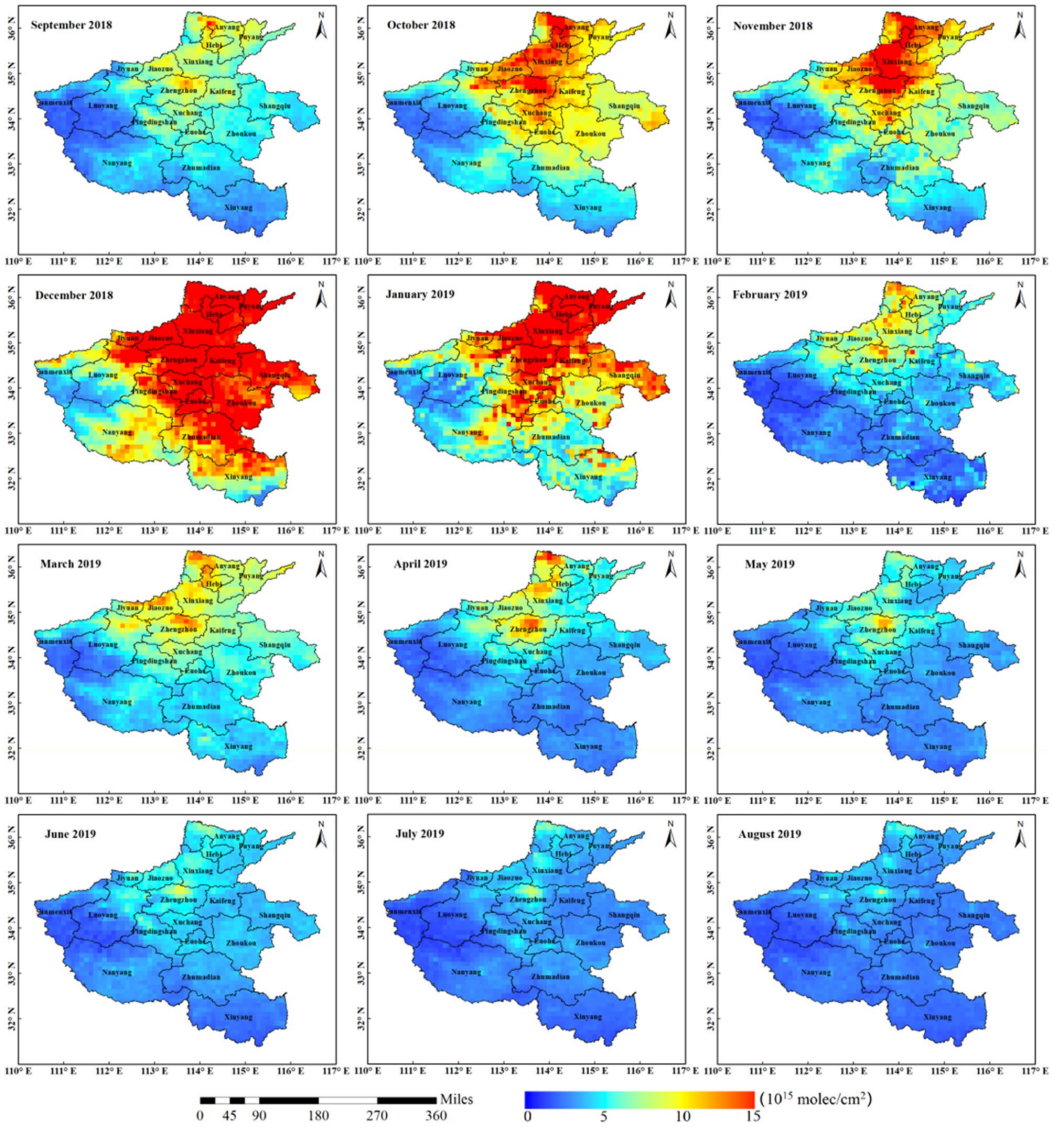
Monthly average distribution of TROPOMI NO_2_ VCDs (× 10^15^ mol/cm^2^) of the COVID-19 early period from September 2018 to August 2019 in Henan Province.

Figure 5 shows the monthly average spatial distribution map of TROPOMI NO_2_ VCDs from September 2018 to August 2019 (the white area is no value). Because VCDs of OMI and TROPOMI have basically the same monthly average variation range, and the above analysis shows that TROPOMI data can describe the regional pollution situation more carefully than OMI data, and TROPOMI sensor is also more advanced than OMI sensor, so there are no longer analyses the monthly average distribution of OMI NO_2_ VCDs, focusing on the monthly average distribution of NO_2_ VCDs of TROPOMI.

From the NO_2_ VCDs of TROPOMI in Figs. 4 and 5, it can be seen that the NO_2_ concentration variation can be divided into two stages as a whole: the rising stage is from September to December 2018, and the declining stage is from January to August 2019. The high concentration range increased from September to October 2018. In November, the range of high concentration was further expanded, the concentration increased in some areas (Zhengzhou, Xinxiang, Anyang and Hebi), and the average concentration of NO_2_ in the whole month decreased compared with that in October. In December 2018 and January 2019, the high concentration area of NO_2_ is more widely distributed than other months, mainly concentrated in the more developed areas in the central and eastern, and north in Henan Province. In February 2019, the troposphere NO_2_ VCDs ranges decreased significantly, mostly concentrated in the northern part of Henan Province adjacent to Shanxi and Hebei. In March 2019, compared with February, the concentration range was expanded and the monthly average concentration increased.

According to the official statistics of Henan Provincial Bureau of Statistics (http://data.stats.gov.cn/ks.htm?cn=E0101), in March 2019, the industrial added value of Henan Province increased by 9%, much higher than other months, which may be part of the reason for the sudden increase of NO_2_ concentration in March. The high concentration range of NO_2_ in April is smaller than that in March, and the high concentration values are mostly found in Anyang, Hebi, Jiaozuo, Xinxiang and Zhengzhou. From May to August, the high value areas in most areas of Henan Province were further reduced, and the high value areas were mostly concentrated in the northern part of Zhengzhou city. On the whole, the variation of tropospheric NO_2_ VCDs is dominated by human emission, which is closely related to human activities, and mostly concentrated in the economically developed and densely populated areas of Henan Province.

### The NO_2_ VCDs spatiotemporal evolution analysis of seasonal variation in early stage of COVID-19 pandemic in Henan province

In order to analyse the seasonal variation characteristics of tropospheric NO_2_ VCDs in Henan Province, according to the seasonal meteorological division method of Henan Province, this paper divides September, October and November 2018 into autumn, December 2018 and January and February 2019 into winter, March, April and May 2019 into spring, June, July and August 2019 into summer. The seasonal average value of the tropospheric NO_2_ VCDs from September 2018 to August 2019 was obtained by the way of monthly average cumulative average (excluding 0 values).

**Figure 6:**
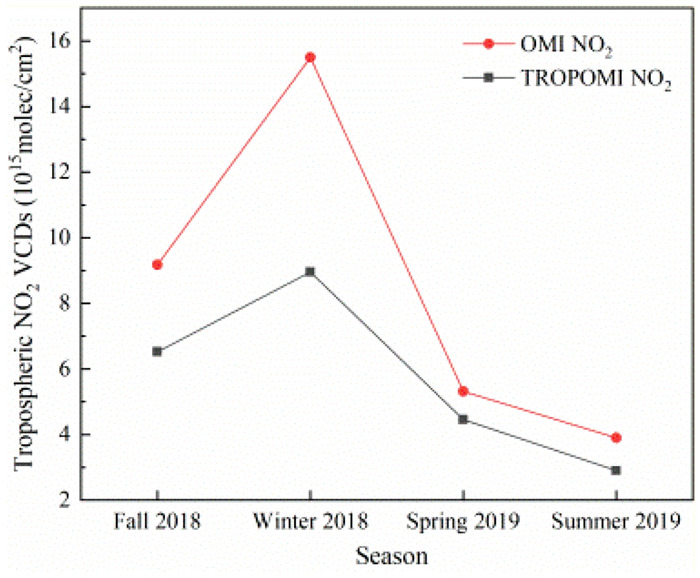
Seasonal variation trend of NO_2_ VCDs of the COVID-19 early period from August 2018 to September 2019 in Henan province.

Figure 6 shows the trend chart of NO_2_ VCDs of OMI and TROPOMI in four seasons. On the whole, they have the same change range in four seasons. The concentration levels are: Winter 2018 > autumn 2018 > spring 2019 > summer 2019. However, the overall NO_2_ VCDs of OMI is higher than TROPOMI, which is the most obvious difference in winter 2018. It is generally believed that the NO_2_ concentration is consistent with season varies for different sensors. However, the difference between the observed waveband and the inversion algorithm will cause concentration result change. It can be seen from Fig. 6 that the tropospheric NO_2_ VCDs is on the high concentration in autumn and winter of 2018. On the one hand, under the influence of low atmospheric temperature and low wind conditions in winter, the chemical reaction of NO_2_ with other components is slow, industrial emissions and automobile exhaust are not easy to diffuse and flow, and the coal-fired emissions generated by heating aggravate the winter in Henan Province; on the other hand, it is also related to the emissions of a large number of gaseous and particulate pollutants into the air during autumn crop harvest. In the spring and summer of 2019, the concentration is relatively low. Although there is burning straw in summer, Henan Province is located in the Central Plains, which belongs to humid and semi-humid, temperate and subtropical of monsoon climate. In summer, it is hot, rainy and windy with strong atmospheric diffusion ability. Strong solar radiation and sufficient rain make the NO_2_ gas in the atmosphere dissipate easily, resulting NO_2_ tropospheric VOCs in summer reaching the lowest value in a year.

**Figure 7:**
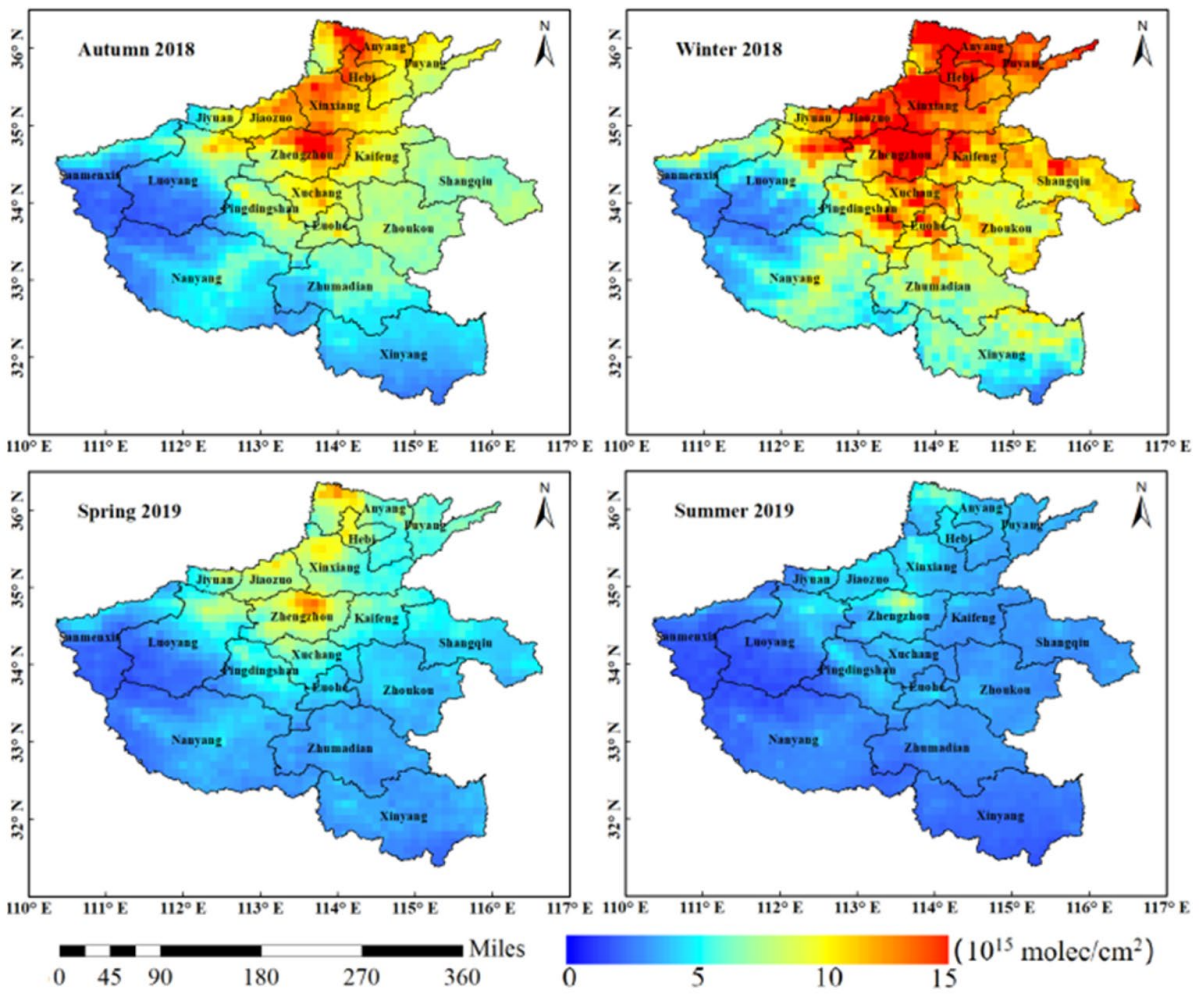
Seasonal mean distribution of TROPOMI NO_2_ VCDs (× 10^15^ mol/cm^2^) of the COVID-19 early period from August 2018 to September 2019 in Henan Province.

In order to better analyse the seasonal spatial variation of the tropospheric NO_2_ VCDs in Henan Province, the seasonal mean distribution of TROPOMI NO_2_ from August 2018 to September 2019 is also given in this paper to reflect the seasonal spatial variation (Fig. 7). Because the seasonal variation trend and the tropospheric NO_2_ VCDs range of OMI and TROPOMI are basically the same, and the observation ability of TROPOMI sensor is much higher than that of OMI sensor, so the seasonal spatial variation of tropospheric NO_2_ VCDs of TROPOMI is selected as the research data. As shown in Fig. 7, the high concentration range of NO_2_ in autumn and winter of 2018 in Henan Province is larger than that in spring and summer of 2019. In autumn 2018, the high NO_2_ concentration range is large, and NO_2_ is concentrated in the central and eastern, and north of Henan. In winter of 2018, NO_2_ concentration in most areas is at high level, increasing from south to north and from west to east. In the spring of 2019, the NO_2_ VCDs is high in the northern part of Henan Province. In the summer of 2019, most areas of NO_2_ are at a low concentration, and the NO_2_ VCDs in the economically developed areas in the north of Zhengzhou and around Anyang is high concentration. This seasonal difference in tropospheric NO_2_ VCDs is mainly related to atmospheric pressure, precipitation, temperature and other meteorological factors as well as anthropogenic emissions.

Specifically, the possible reasons for the seasonal variation of troposphere NO_2_ VCDs are:

1. In autumn and winter, the low temperature, the dry air and the control of continental high in most areas of China jointly led to the continuous accumulation of NO_2_ pollutants. The reason is that the air mobility in the near ground layer is weak in autumn and winter, and pollutants are easy to accumulate, which is not conducive to diffusion^48–50^.
2. In spring and summer, with the increase of solar radiation, temperature and rainy, the NO_2_ concentration will be diluted. Under the condition of constant anthropogenic emission source, the chemical reaction consume troposphere NO_2_ VCDs, resulting in the decrease of NO_2_ concentration.

### The NO_2_ VCDs spatiotemporal evolution analysis of annual average in early stage of COVID-19 pandemic in Henan province

**Figure 8:**
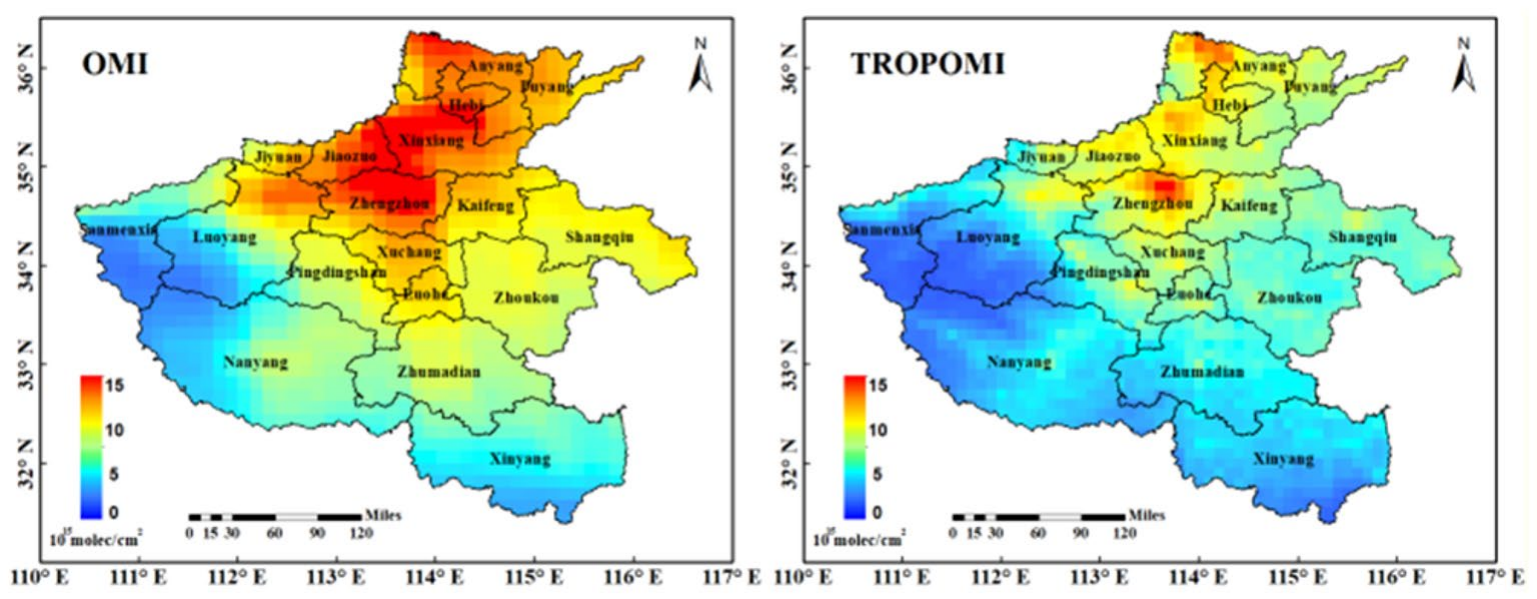
Annual mean distribution of tropospheric NO_2_ VCDs (× 10^15^ mol/cm^2^) of the COVID-19 early period from September 2018 to August 2019 in Henan Province.

In order to analyse the spatial variation of troposphere NO_2_ VCDs in Henan Province, the annual mean value of troposphere NO_2_ VCDs from September 2018 to August 2019 was obtained by accumulating the monthly mean NO_2_ VCDs of OMI and TROPOMI (excluding 0 values). It can be seen from Fig. 8 that the high and low concentration distribution areas of NO_2_ in Henan Province are roughly the same, but the NO_2_ VCDs of TROPOMI is lower and more detailed than OMI. As a whole, the concentration range of NO_2_ is from south to north and from west to east with increasing trend. The reasons for this trend may be:

#### It is related to the distribution of economic conditions

Most of the economically developed areas in the province are concentrated in the northern and eastern areas with Zhengzhou as the centre. These areas are mainly light industry and large-scale power plants. They are the economic development centre of the province, with a large population density, which together lead to the increase of NO_2_ concentration in these areas.

#### It has a lot to do with the terrain

The annual distribution map of NO_2_ VCDs of OMI and TROPOMI (Fig. 8) shows that the high value areas of the whole province are distributed in the north of Zhengzhou, the northwest of Xinxiang, the northwest of Kaifeng, Jiaozuo, Jiyuan, Hebi and the north of Anyang and other economically developed plain areas. The distribution map of NO_2_ VCDs of TROPOMI shows that the NO_2_ concentration in one area in the north of Zhengzhou is on the high side, which is in Erqi District, Jinshui District, Zhongyuan District and Guancheng Hui District with relatively developed economy. This area is an important transportation hub in China, including trains, subways, automobiles and other important means of transportation in the country; while the low value areas are all distributed in the mountainous areas of southwest Henan, which is closely related to the high vegetation coverage and less population living in the mountainous areas.

**Figure 9:**
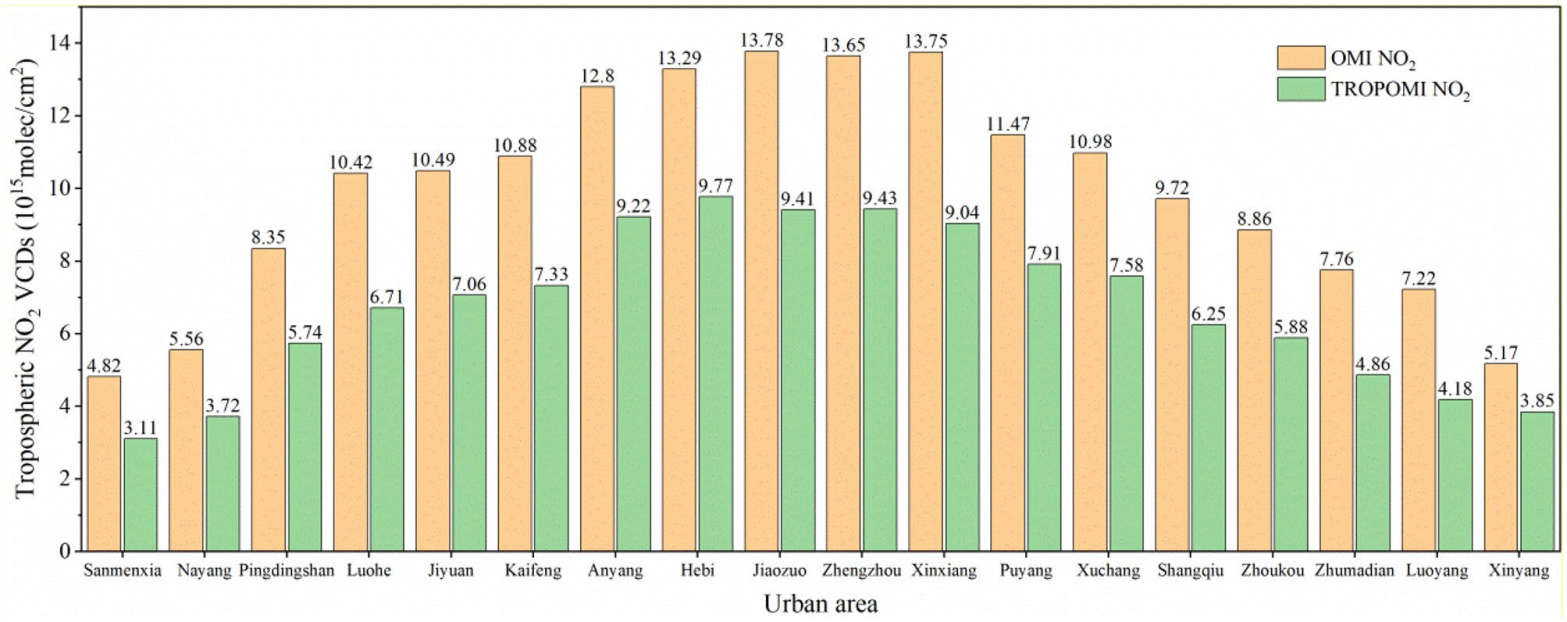
Annual average variation of tropospheric NO_2_ VCDs (× 10^15^ mol/cm^2^) in 18 cities of the COVID-19 early period from September 2018 to August 2019 in Henan Province.

Figure 9 shows the annual mean changes of tropospheric NO_2_ VCDs in 18 urban areas of Henan Province from August 2018 to September 2019. As shown in Fig. 9, the high and low NO_2_ VCDs of TROPOMI and OMI are roughly the same. The high NO_2_ concentration cities are mainly in Xinxiang, Anyang, Jiaozuo, Zhengzhou, Hebi and other developed and densely populated northern and eastern areas of Henan Province. Among them, NO_2_ VCDs of TROPOMI shows that the NO_2_ concentration in Hebi City is the highest, reaching 9.77 × 10^15^ mol/cm^2^. This is because Hebi is a thermal power base in Henan Province, which is a city, built on coal, and there are many enterprises with large power consumption such as cement and metallurgy. However, NO_2_ VCDs of OMI showed that the highest NO_2_ concentration in Jiaozuo was 13.78 × 10^15^ mol/cm^2^; the low concentration is mainly in Sanmenxia, Nanyang City, Xinyang, Luoyang, Zhumadian and other mountainous and hilly areas, mainly these areas are located in the underdeveloped mountainous areas, with small population density, less NO_2_ pollution sources from vehicles and factories, and small NO_2_ pollution in neighbouring Shaanxi Province and Hubei Province, which results in low NO_2_ concentration.

### The TROPOMI NO_2_ VCDs spatiotemporal evolution analysis of YoY rate before and after COVID-19 pandemic in Henan province

Since large number of social and economic activities have been suspended on lockdown to prevent and control the COVID-19 pandemic outbreak, which also provides a good opportunity for the scientific research of air pollution. Because TROPOMI sensor has higher observation accuracy and resolution than OMI sensor, the following only uses TROPOMI data to analyse the spatiotemporal evolution of NO_2_ pollution in Henan Province. Specifically, the temporal and spatial variation of NO_2_ VODs in 18 urban areas of Henan Province during the previous year of pandemic period (from January 21, 2019 to February 20, 2019), the previous month of pandemic period (from December 21, 2019 to January 20, 2020), and the COVID-19 pandemic period (from January 21, 2020 to February 20, 2020) were analysed.

**Figure 10:**
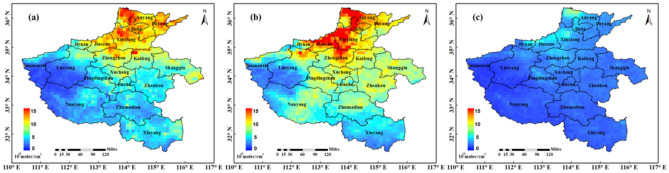
Spatial distribution of tropospheric NO_2_ VCDs of TROPOMI (× 10^15^ mol/cm^2^) in Henan Province: (**a**) Previous year of pandemic period (from January 21, 2019 to February 20, 2019); (**b**) Previous month of pandemic period (from December 21, 2019 to January 20, 2020); (**c**) COVID-19 pandemic period (from January 21, 2020 to February 20, 2020).

In order to avoid the impact of the resume to work of the pandemic on the NO_2_ concentration, we select the daily products of NO_2_ VCDs of TROPOMI from January 21, 2020 (2 days before the lockdown of Wuhan City) to February 20, 2020 (COVID-19 pandemic period) for one month, and analyse the spatial distribution of NO_2_ during the pandemic in Henan Province. The COVID-19 pandemic can be seen as a great influence on NO_2_ emissions in Henan province from Fig. 10c. Except for Anyang, Xinxiang, Jiaozuo and Jiyuan in the north of Henan Province, the NO_2_ VCDs in other areas was generally lower than 5 × 10^15^mlec/cm^2^. Among them, the NO_2_ emission in the high concentration area mainly comes from the electric power, transportation and other industries, as well as the emission of bulk coal combustion and the transportation of advection layer caused by heating in winter^51^. According to the statistics of the average value of each urban area (Table 1), there are only Jiaozuo, Anyang, Hebi, Jiyuan, Xinxiang and Zhengzhou in Henan Province, with NO_2_ VCDs more than 3 × 10^15^ mol/cm^2^. The NO_2_ VCDs are low in other urban areas. The lowest of NO_2_ VCDs are Sanmenxia, and it is 1.41 × 10^15^ mol/cm^2^.

**Table 1:** The NO_2_ VCDs of COVID-19 pandemic period in 18 urban areas of Henan Province.

City	Average NO_2_ VCDs (× 10^15^ mol/cm^2^) previous year of pandemic period (21-01-2019 to 20-02-2019)	Average NO_2_ VCDs (× 10^15^ mol/cm^2^) previous month of pandemic period (21-12-2019 to 20-01-2020)	Average NO_2_ VCDs (× 10^15^ mol/cm^2^) during pandemic period (21-01-2020 to 20-02-2020)	YoY rate(%) between (21-01-2019 to 20-02-2019) and (21-01-2020 to 20-02-2020)	MoM rate(%) between (21-12-2019 to 20-01-2020) and (21-01-2020 to 20-02-2020)
Anyang	11.57	12.23	**3.79**	− 67.22	− 69.01
Hebi	**12.43**	13.48	3.48	− 71.99	− **74.17**
Jiyuan	7.56	8.70	3.34	− 55.81	− 61.59
Jiaozuo	10.21	**14.15**	3.88	− 62.02	− 72.61
Kaifeng	7.91	8.77	2.56	− 67.60	− 70.80
Luoyang	3.27	5.10	1.69	− 48.37	− 66.91
Luohe	5.33	6.92	2.16	− 59.45	− 68.81
Nayang	**3.00**	5.36	**1.43**	− 52.48	− 73.38
Pingdingshan	4.27	7.36	2.21	− **48.23**	− 69.99
Puyang	11.56	9.67	2.83	− 75.48	− 70.68
Sanmenxia	2.61	4.09	1.41	− 46.10	− 65.60
Shangqiu	6.94	7.83	2.18	− 68.53	− 72.09
Xinxiang	10.90	12.46	3.34	− **69.37**	− 73.20
Xinyang	4.61	**3.99**	1.82	− 60.51	− **54.33**
Xuchang	6.67	8.24	2.43	− 63.60	− 70.54
Zhengzhou	8.34	10.00	3.02	− 63.83	− 69.81
Zhoukou	5.00	7.71	2.21	− 55.80	− 71.34
Zhumadian	3.92	6.07	1.87	− 52.35	− 69.22

The bold numbers represent the maximum and minimum values of the values in each column.

Figure 10a shows the NO_2_ VCDs distribution of Henan Province in previous year of pandemic period (from January 21, 2019 to February 20, 2019). It can be find from Fig. 10a that the NO_2_ VCDs is generally high in the same period of 2019, and the distribution range is also wider. Especially, the NO_2_ VCDs is close to or more than 10 × 10^15^ mol/cm^2^ in Hebi, Anyang, Puyang, Xinxiang, Jiaozuo, Zhengzhou and other densely populated plain areas in the northeast of Henan Province. However, the NO_2_ VCDs in these areas decreased significantly during the pandemic period, which shows that the impact of the pandemic on the NO_2_ VCDs in high concentration areas are more obvious.

The YoY rate of NO_2_ VCDs in different areas are calculated and analysed by Formulas (12) and (15) of SI-APSTE model. Results as shown in Table 1, the YoY rate (decline rate) of each urban area is very large, with a decline rate of more than 46%. Both Puyang and Hebi decreased by more than 70%, with a decrease rate of 75.48% and 71.99%, respectively. The main reasons for these obvious reductions are as follows: on the one hand, the concentration in these areas is high at ordinary times, and the reduction space is large; on the other hand, it may be caused by the strict control measures during the pandemic period, the shutdown of factories and the lack of people's travel.

### The TROPOMI NO_2_ VCDs spatiotemporal evolution analysis of MoM rate before and after COVID-19 pandemic in Henan province

Figure 10b shows the spatial distribution of NO_2_ in the previous month of pandemic period (from December 21, 2019 to January 20, 2020). The NO_2_ VCDs spatial distribution of previous month of pandemic period (Fig. 10b) is similar to that previous year of pandemic period (from January 21, 2019 to February 20, 2019) (Fig. 10a). However, the NO_2_ concentration of previous month of pandemic period is wider and higher. The NO_2_ VCDs in the northeast of the province is more than 10 × 10^15^ mol/cm^2^. Compare with the NO_2_ spatial distribution of previous month of pandemic period (Fig. 10b) and COVID-19 pandemic period (from January 21, 2020 to February 20, 2020) (Fig. 10c), the scope of NO_2_ in the whole province has decreased to a large extent. The scope has become smaller and the concentration has become lower. It can be seen that the decrease of NO_2_ in Henan Province during the pandemic period is very large, which may be the reason for people's home isolation, factory shutdown and so on. The specific influencing factors are greater need to be further studied.

The MoM rate of NO_2_ VCDs in different areas are calculated and analysed by Formulas (12) and (14) of SI-APSTE model. Results as shown in Table 1, the MoM rate (decline rate) of the whole province is more than 54%. Henan Province has always been one of the provinces with powerful governance in the whole country during the pandemic period. The measures for pandemic control are strictly controlled, and the pandemic control time is relatively early, so the effect is very significant. In the whole province, 9 urban areas, including Hebi, Nanyang, Xinxiang, Jiaozuo, Shangqiu, Zhoukou, Kaifeng, Puyang and Xuchang, account for more than 70% of the MoM rate. Among them, Hebi saw the largest decline, reaching 74.17%. From the month before the pandemic to the pandemic period, the decrease of NO_2_ VCDs was so significant; this indicated that human activities were very important factors affecting NO_2_ concentration.

## Conclusion

This paper describes the complex dynamic system of air pollution based on cellular automata and restricted agents, the SI-APSTE model is proposed based on swarm intelligence and spatiotemporal sampling theorem, and further deduces the spatiotemporal evolution analysis method of air pollution. In order to verify the effectiveness of the SI-APSTE model, taking the NO_2_ pollution before and after the COVID-19 pandemic in Henan Province as an example, based on the SI-APSTE model, the NO_2_ VODs products of TROPOMI and OMI were compared and analysed, and the temporal and spatial distribution characteristics of the tropospheric NO_2_ VCDs from August 2018 to September 2019 in Henan Province were studied. Using TROPOMI data, the spatiotemporal variation of NO_2_ VODs in 18 urban areas of Henan Province were studied in the COVID-19 pandemic periods, the previous year of pandemic periods and the previous month of pandemic periods.

This research shows that the SI-APSTE model can effectively implement the spatiotemporal evolution analysis for NO_2_. The model can also be used to study the temporal and spatial evolution of other trace air pollutants such as other nitrogen oxides, sulfur oxides, carbon oxides, hydrocarbons and ozone. Making full use of the characteristics of environmental big data and SI algorithm, the SI-APSTE model would deeply reveal the mechanism of air pollution, and also establish a theoretical basis for pollution sources identification and trend prediction of air pollution.
